# Supplementary material for: Identification of photoperiod-induced specific miRNAs in the adrenal glands of Sunite sheep (Ovis aries)
Source: Front Vet Sci. 2022 Jul 22;9:888207. doi: 10.3389/fvets.2022.888207 (PMC9354845; doi:10.3389/fvets.2022.888207)
Supplement: Supplementary file 1 [file Data_Sheet_1.ZIP › Supplementary file/miRDeep2 file.zip!/miRDeep2 file/Novel_Structure/SP42A1/Novel_621.pdf]

Provisional ID	:	NC_019471.2_17498
Score total	:	89.8
Score for star read(s)	:	3.9
Score for read counts	:	81.7
Score for mfe	:	2.6
Score for randfold	:	1.6
Score for cons. seed	:	
Total read count	:	172
Mature read count	:	169
Loop read count	:	0
Star read count	:	3

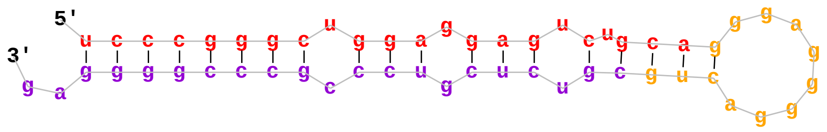

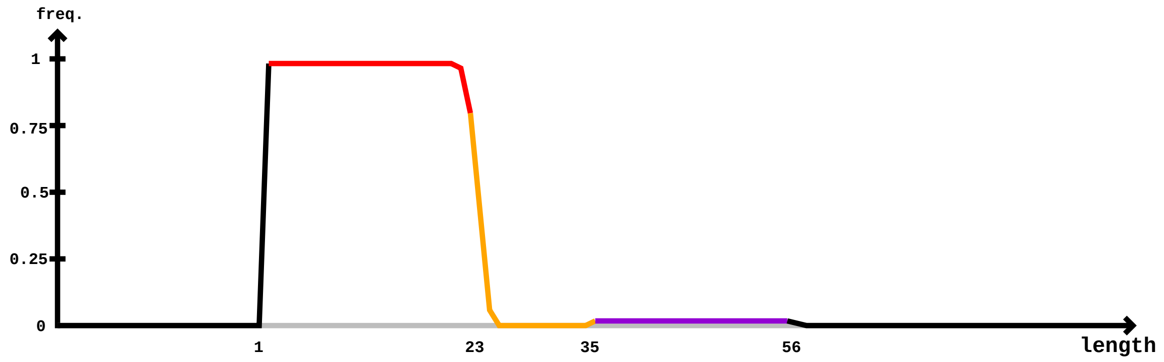

Mature **Star**

```
5'- ggaccugggcaggggcggggucccgggcuggaggagucugcagggaggggacugcgucucgucccgcccggggagaccgccccuugccgcucccaggacgguggauagcg   -3'    obs
    ggaccugggcaggggcggggucccgggcuggaggagucugcagggaggggacugcgucucgucccgcccggggagaccgccccuugccgcucccaggacgguggauagcg          exp
    .......((((((((((..((((((((.(((.(((.(.((((........))))).))).))).))))))))...))))))).))))(((.(((......)))..))).     reads    mm    sample
    ....................ucccgggcuggaggagucug......................................................................     3        0     seq
    ....................ucccgggcuggaggagucugc.....................................................................     29       0     seq
    ....................ucccgggcuggaggagucugca....................................................................     83       0     seq
    ....................ucccgggcuggaggagucugcag...................................................................     44       0     seq
    ....................ucccgggcuggaggagucugcagg..................................................................     10       0     seq
    ......................................................cgucucgucccgcccggggag...................................     3        0     seq
```
